# Single-incision laparoscopic surgery

**DOI:** 10.4103/0972-9941.72356

**Published:** 2011

**Authors:** Alfred Cuschieri

**Affiliations:** Institute for Medical Science and Technology, University of Dundee, Scotland and Scuola Superiore Sant’Anna di Studi Universitari, Pisa, Italy

This issue of JMAS is dedicated to a variant of the well-established multiport minimal access surgery, the advent of which in the mid-1980s revolutionised surgical practice and care beyond the dreams of its pioneers. As illustrated in Figure 1, the adoption of the MAS approach drastically reduced the traumatic insult to the patient not just by minimizing the access trauma inherent to traditional open surgery but equally important, though often overlooked, by safeguarding the internal environment of the patients’ organs (the milieu intérieur described by the great physiologist, Claude Bernard). It is, nowadays, equated with homeostasis such that the gastrointestinal tract is no longer exposed to the operating room air with the attendant dual risk of desiccation and absorption off endotoxin present in the operating room air by the peritoneal and serosal lining. The benefits that have been accrued as a result of the widespread adoption of the MAS approach are well-established but beyond the scope of this editorial except to emphasize that the MAS approach is now the gold standard for many common and advanced operations across the surgical specialties.

**Figure 1:**
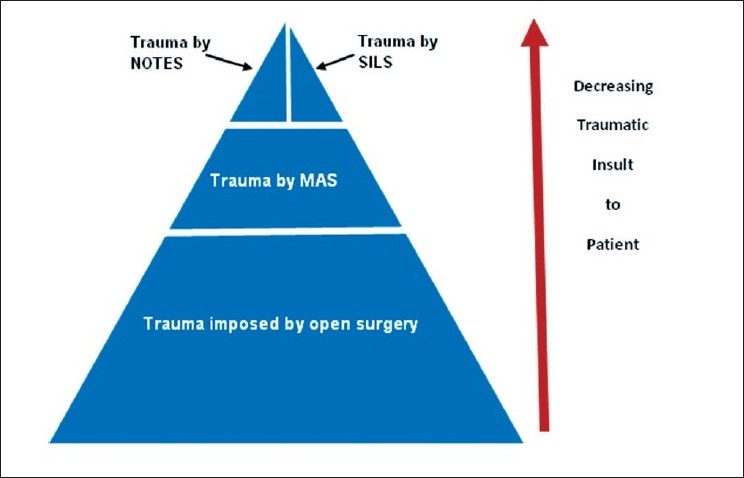
Schematic representation of the reduction in trauma of access to the patients as a consequence of the adoption of MAS and variant MAS approaches.

## NOTES VS. SILS

In recent years, two variants of the MAS approach have been introduced in the quest for further abrogation of the traumatic insult to the patient, thereby further reducing the postoperative pain and visible external scars [Figure 1]: natural orifice transluminal endoscopic surgery (NOTES) and single-incision laparoscopic surgery (SILS) also referred to, regrettably by a variety of eponymous and unscientific names. In essence, there is at the moment an apparent conflict between these two variant MAS approaches which appears to extend beyond the surgical profession to industry in respect of the underpinning technologies: flexible and rigid optics/instrumentation. As usual in medical science, the truth lies somewhere between these opposing and entrenched views. The situation is clarified to some extent if one addresses the matter objectively and in scientific terms. Thus all the various NOTES interventions can and should be classed in scientific terms as:

- *Hollow-visceral transperitoneal (HVT)*: transgastric, transoesophageal, transcolonic, trans vesical – access to the peritoneal cavity by planned perforation of a hollow viscus.
- *Squamous conduit intraperitoneal (SCI)*: transvaginal, transanal – direct access to the peritoneal cavity

These two categorise of the NOTES approach are, from the surgical perspective, fundamentally different one from the other in three respects. Thus, whereas HVT imposes a new operating paradigm and requires new interventional flexible technologies which is still in development, it introduces an extra layer of risk (intentional perforation of an intraperitoneal hollow organ), which SCI does not. Thirdly, SCI is performed with existing laparoscopic instrumentation and rigid optics. In many respects, the transvaginal approach approximates to vaginal SILS and the transanal approach (still in its infancy) is a logical extension of transanal endoscopic microsurgery pioneered by G Buess. This may well explain the very limited reported cases with no large series by the HVT in contrast to the substantive series of laparoscopic cholecystectomy (LC) by the SCI (transvaginal) approach. Even so to date, the transvaginal approach has been overshadowed by abdominal SILS.

## SINGLE-INCISION LAPAROSCOPIC SURGERY

**Figure 2:**
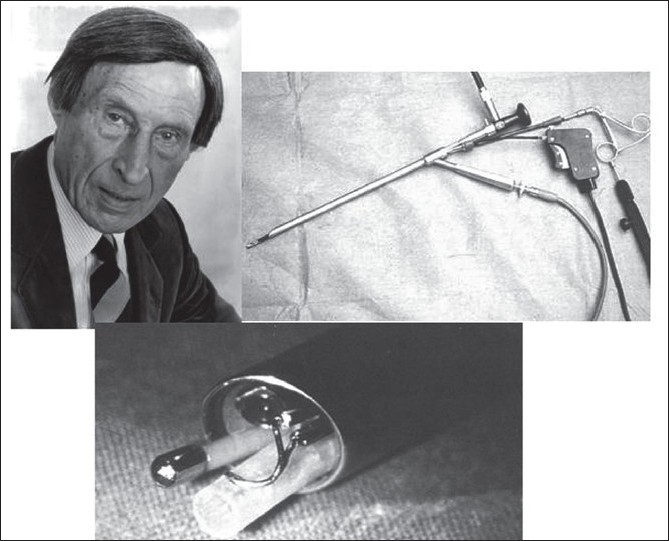
Dr Raimund Wittmoser and the multifunctional port he developed and used for thoracoscopic surgery on the sympathetic chain.

The concept of SILS is not new and has to be attributed to the late Dr. Raimund Wittmoser, the father of modern thoracoscopic surgery of the autonomic nervous system [Figure 2]. R Wittmoser used a single-intercostal incision through which he inserted a multifunctional port which contained all the instruments including the optic [Figure 2] for all his operations on the sympathetic and parasympathetic he performed as early as the mid-1970s.

Without doubt, SILS has had the greatest uptake in routine surgical practice and more than 1,100 LCs have been published to date by this approach with a reported conversion rate of 6%. What is equally important is that SILS has been used for a wide spectrum of advanced laparoscopic operations across the surgical specialties: colorectal resections, bariatric operations, nephrectomies including living-related donor kidney harvest, splenectomies etc. To date 137 colorectal resections have been reported, 60% of these being resections for cancer. The overall conversion rate to conventional multiport laparoscopic surgery or laparotomy has been surprisingly low at less than 4% with a very respectable morbidity (7%) compared to conventional multiport laparoscopic colorectal surgery. Additionally, it appears from the reported series pertaining to resections for colonic cancer that these were oncologically appropriate, certainly with respect of resection margins.

## TECHNOLOGIES FOR SILS

**Figure 3:**
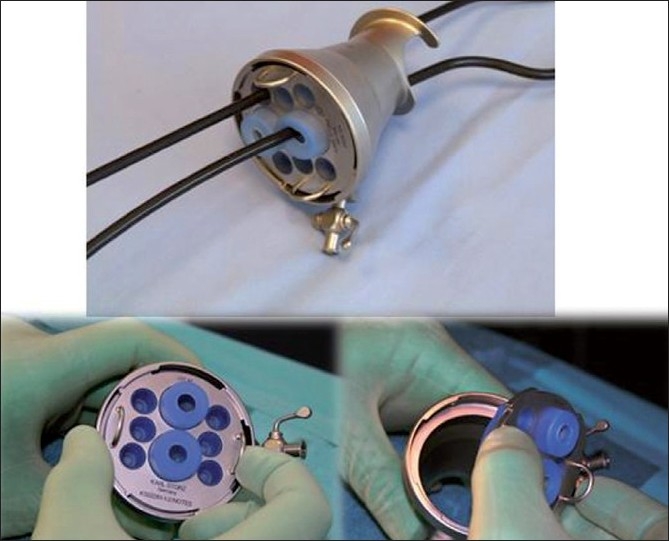
Dundee Endocone with detachable cap (Bulkhead) containing eight valved inlets admitting 5-15 mm instruments (includingg staplers).

The technology for SILS has advanced at an accelerated pace in recent years. All the established instrument companies have produced or market a SILS port and new companies, e.g., SurgiQuest have emerged specializing in novel port systems. Regrettably, however, there have been no comparative studies on the relative performance in terms of triangulation possible and ease of use between these SILS ports, the vast majority of which are disposable. Currently, there are only two reusable SILS ports available: the X-port (Storz, Tuttlingen, Germany) and the EndoCone designed and developed at the Institute for Medical Science and Technology (IMSaT) in Dundee in association with Storz. The EndoCone has a detachable bulk head (cap) with contains six lateral valved inlets admitting use of 5-mm instruments and two central inlets which allow the insertion of large instruments including staplers [Figure 3]. The surgeon is able to use three instruments and an optic at any one time during the course of the operation.

**Figure 4:**
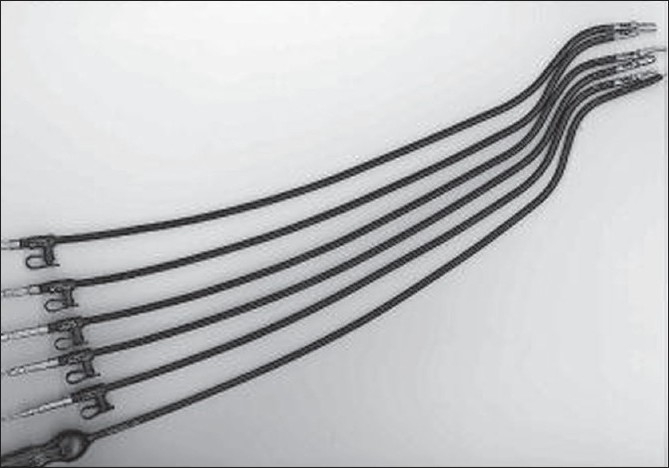
Storz Tuttlingen - curved coaxial instruments designed at IMSaT, Dundee.

**Figure 5:**
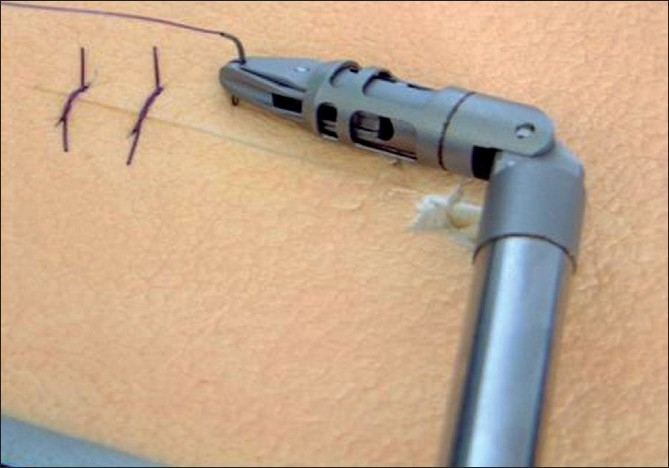
The 5-mm DARES handheld manipulator with controlled flexion of the distal segment and rotation of the jaws providing 6 degrees of freedom.

The instrumentation for SILS has also progressed significantly in the last few years with the development of proximally deviating curved coaxial instruments which enable limited triangulation and retraction without the needs for retraction/suspension sutures [Figure 4]. Intracorporeal suturing during SILS will be greatly facilitated with the introduction of 5-mm hand-held surgical manipulators with six degrees of freedom. These enable graded flexion of the distal segment of the instrument and through the incorporation of an internal wrist enabling rotation of the tip and jaws, greatly facilitate both continuous and interrupted suturing [Figure 5].

### Outstanding issues concerning SILS

There is no doubt that there has been significant progress and uptake of SILS by surgeons as a result of the substantial advances in port technology and related instrumentation which has enabled performance of a range of laparoscopic operations across the surgical specialties. Equally, the published experience has confirmed the safety and efficacy of the SILS approach. However, there are a number of important issues which need to be addressed before we can confirm with good evidence that this variant laparoscopic approach does indeed benefit the patient. Thus aside from reduction of visible scars, the jury remains out on whether SILS does indeed reduce postoperative pain and adhesion formation. Likewise the contraindications to SILS have yet to be clarified and defined. There is one potential area of concern and this relates to an increased risk of incisional periumbilical hernia formation. The extent of this perceived complication will only be established by prospective cohort or randomised studies and longer follow-up. Meantime, we need to ensure that these umbilical wounds are closed with a meticulous technique using non-absorbable material.

Even with the best instrumentation currently available, the SILS approach imposes restrictions on instrument manipulation, retraction and limits triangulation. The full potential of this variant approach may only be achieved by the adoption of robotic master slave manipulators. Indeed major operations (radical prostatectomy, dismembered pyeloplasty, radical nephrectomy, etc.) have already been reported with the existing DaVinci robot and the US company, SurgiQuest, Inc is designing a custom SILS port for Intuitive Surgical based on SugiQuest’s AirSeal single port access. In Europe on major funding from the EU Framework 7 project, Prof Paolo Dario and A Cuschieri are coordinating a large consortium concerned with the development of a low-cost dedicated system for robotic SILS-known as the ARAKES IP project. The ARAKNES SILS robot is currently at the prototype stage and will be ready for animal testing in 18 months time.

